# Role of Vitamin K in Chronic Kidney Disease: A Focus on Bone and Cardiovascular Health

**DOI:** 10.3390/ijms23095282

**Published:** 2022-05-09

**Authors:** Federica Bellone, Maria Cinquegrani, Ramona Nicotera, Nazareno Carullo, Alessandro Casarella, Pierangela Presta, Michele Andreucci, Giovanni Squadrito, Giuseppe Mandraffino, Marcello Prunestì, Cristina Vocca, Giovambattista De Sarro, Davide Bolignano, Giuseppe Coppolino

**Affiliations:** 1 Department of Clinical and Experimental Medicine, University of Messina, I-98100 Messina, Italy; federica.bellone@unime.it (F.B.); mariacinquegrani@gmail.com (M.C.); giovanni.squadrito@unime.it (G.S.); giuseppe.mandraffino@unime.it (G.M.); 2 Azienda Sanitaria Provinciale di Catanzaro, I-88100 Catanzaro, Italy; ramona.nicotera@gmail.com (R.N.); prunestim@gmail.com (M.P.); 3 Department of Health Sciences, “Magna Graecia” University, I-88100 Catanzaro, Italy; nazareno.carullo@gmail.com (N.C.); al.cas1993@gmail.com (A.C.); piera.presta@gmail.com (P.P.); andreucci@unicz.it (M.A.); cristina.vocca@studenti.unicz.it (C.V.); desarro@unicz.it (G.D.S.); dbolignano@unicz.it (D.B.)

**Keywords:** kidney, vitamin K, phylloquinone, menaquinone, cardiovascular disease, calcification, hypertension, osteoporosis, bone, fracture

## Abstract

Chronic kidney disease (CKD) is commonly associated with vitamin K deficiency. Some of the serious complications of CKD are represented by cardiovascular disease (CVD) and skeletal fragility with an increased risk of morbidity and mortality. A complex pathogenetic link between hormonal and ionic disturbances, bone tissue and metabolism alterations, and vascular calcification (VC) exists and has been defined as chronic kidney disease–mineral and bone disorder (CKD-MBD). Poor vitamin K status seems to have a key role in the progression of CKD, but also in the onset and advance of both bone and cardiovascular complications. Three forms of vitamin K are currently known: vitamin K1 (phylloquinone), vitamin K2 (menaquinone), and vitamin K3 (menadione). Vitamin K plays different roles, including in activating vitamin K-dependent proteins (VKDPs) and in modulating bone metabolism and contributing to the inhibition of VC. This review focuses on the biochemical and functional characteristics of vitamin K vitamers, suggesting this nutrient as a possible marker of kidney, CV, and bone damage in the CKD population and exploring its potential use for promoting health in this clinical setting. Treatment strategies for CKD-associated osteoporosis and CV disease should include vitamin K supplementation. However, further randomized clinical studies are needed to assess the safety and the adequate dosage to prevent these CKD complications.

## 1. Introduction

Chronic kidney disease (CKD) is characterized by simultaneous vascular calcifications and impaired bone metabolism [1]. Particularly, an imbalance of the bone–vascular axis with consequent alterations of both vascularization and bone have been demonstrated [2]. Even though the mechanistic link of this crosstalk between the vascular and skeletal system is poorly understood so far, some hormones, including parathyroid hormone (PTH) and 1,25-dihydroxy vitamin D3, are acknowledged to orchestrate both skeletal and vascular mineralization as well as stem cell regeneration [3]. Therefore, the term “calcification paradox” was coined to indicate the association of ectopic mineralization in the vasculature with impaired bone turnover and decreased bone mineral density (BMD) [4]. In the last years, the knowledge about the key role of vitamin K has exponentially increased, due to its well-recognized involvement in vascular calcifications, cardiovascular disease, and bone tissue impairment. Recently, growing evidence seems to suggest that vitamin K supplementation could be a tool to prevent the rapid progression of vascular calcifications and to preserve bone health in CKD patients [5]. In this context, we aimed to focus on the current knowledge on vitamin K biological functions, its involvement in the relationships among cardiovascular disease (specifically in hypertensive patients) and bone metabolism in CKD patients, and the potential use of Vitamin K vitamers for promoting health in this clinical setting.

## 2. Methods

### Search Strategy

A scoping review of the available literature was conducted. Firstly, the studies were retrieved from the online databases PubMed, Scopus, and Web of Knowledge, by matching the following keywords: “chronic kidney disease”, “vitamin K”, “vascular calcification”, “bone metabolism”, “osteoporosis”, and “cardiovascular disease”. A preliminary filter on the online search was applied by language (English) and availability of full text articles. Additionally, the reference lists of the included studies were examined in order to identify further potentially relevant studies missed during the database search. The online search was definitively completed on 15 March 2022.

## 3. Vitamin K: Chemistry, Nutritional Sources, Distribution and Metabolism

The term vitamin K, or naphthoquinone, refers to a family of fat-soluble molecules which have a similar structure made by a 2-methyl-1,4-naphthoquinone ring but with different origin and function. Currently, three primary forms are known, defined as vitamers, which differ in the side chains linked to the 2-methyl-1,4-naphthoquinone ring at the position 3 [6]; namely, these are vitamin K1 (phylloquinone), vitamin K2 (menaquinone), and vitamin K3 (menadione). The main known biological function of vitamin K1 is played in blood clotting, since it acts as a cofactor for the enzymatic conversion of glutamic acid (Glu) residues to gamma-carboxyglutamic acid (Gla) in vitamin K-dependent proteins (VKDPs), through vitamin K-dependent gamma-glutamyl carboxylase, localized in the endoplasmic reticulum of the cells of all mammalian tissues [7,8,9], and for the conversion of protein-bound glutamate in carboxy-glutamate, needed for II, VII, IX, and X coagulation cascade factors, and for the natural anticoagulants proteins S and C [10,11]. The source of vitamin K1 is mainly represented by leafy or flowering vegetables (spinach, lettuce, broccoli, cabbage, Brussels sprouts, turnip greens), but chickpeas, peas, soya, green tea, eggs, pork, and beef liver also contain vitamin K1 [12]. Vitamin K2 is synthetized essentially by intestinal microbiota and is denoted as menaquinone (MK); according to the length of the isoprene chain attached to the methylated naphthoquinone ring, several different forms could be identified, as numbered from 4 to 13. MK-4 is obtained from the conversion of phylloquinone or menadione and is found mainly in meat and animal by-products such as eggs, cow’s milk and yoghurt [13,14,15]. On the other hand, MK-7 is a long-chain form also produced by intestinal bacteria and it is found in fermented food, such as cheese and soya [16]. The MK4 and MK7 are two of the most common menaquinones in the human diet, along with MK8, MK9, and MK10 [13]. Vitamin K3, also known as menadione, was formerly considered to be a synthetic form of vitamin K. However, it has been demonstrated that vitamin K3 could also originate in the intestine as the intermediate product of oral vitamin K1 conversion to vitamin K2, namely MK4 [17,18].

Vitamin K absorption occurs in different tracts of the intestine: vitamin K1 is absorbed in the ileum; vitamins K2 in the colonic portions. Efficient biliary and pancreatic function is essential for its adequate absorption. Vitamin K molecules are incorporated into chylomicrons and then released to very low-density lipoprotein (VLDL) and low-density lipoprotein (LDL), with subsequent release to tissues. Vitamin K1 and K2 should be continually synthetized and supplied by intestinal bacteria, due to their relatively short half-life (17 h). The catabolism of vitamin K1 and of vitamin K2 shares common mechanisms, beginning with initial hydroxylation mediated by CYP4F2, followed by shortening of the polyisoprenoic side chain via b-oxidation to carboxylic acids (in 5 C, 7 C, or 10 C metabolites), which are glucuronidated and excreted in urine and bile [7,19,20].

In healthy people, fasting plasma phylloquinone concentrations have been reported to range from 0.29 to 2.64 nmol/L [21]. However, to evaluate serum levels of vitamin K is difficult to perform, as they are influenced by several factors (e.g., low plasma levels, non-polar nature, and lipid interference). Diet and inflammation are additional variables influencing the plasma levels. Therefore, vitamin K levels are often estimated indirectly by measuring prothrombin time (for vitamin K1) or the concentration of decarboxylated γ-carboxyglutamic acid (Gla) proteins (not available for all laboratories) [22,23].

The intake recommendations for vitamin K by the World Health Organization (WHO) and the Food and Agriculture Organization (FAO) are 65 mcg/day for men and 55 mcg/day for women, based on a calculated requirement of 1 mcg/day/kg body weight. The Italian Society of Human Nutrition (SINU) recommended an age-stratified intake of vitamin K: 140 mcg/day or 170 mcg/day for people aged 18–59 and >60-years, respectively [24]. Vitamin K deficiency is correlated with increased rate of cardiovascular events [7]. Observational studies showed an inverse relationship between vitamin K2 and vascular calcifications (VC) [25], while vitamin K1 intake was not significant [26].

Notwithstanding, Xu et al., recently analyzed prospective clinical trials involving CKD people with the aim to investigate which kind of intervention could attenuate VC, evaluated through radiologic methods. They concluded that conflicting data exist regarding vitamin K therapy in CKD and VC progression [27]. Vitamin K is also important for the regulation of the glycemic status by reducing the risk of developing diabetes mellitus and improving insulin sensitivity. Vitamin K2, on the other hand, plays a predominant role in bone development, vascular protection, metabolic, liver and renal diseases. In this regard, vitamin K2 is needed for the synthesis of osteocalcin in the bone and matrix GLa protein in the cartilage and in the blood vessels wall. Thus, it plays a pivotal role in calcium transport, preventing calcium deposition in blood vessels and calcium mobilization from bone tissues [11].

## 4. Vitamin K and Chronic Kidney Disease

Chronic kidney disease (CKD) patients are characterized by poor vitamin K status [28]. Multiple factors can affect vitamin K stores in CKD patients, and the main causes of its deficiency include food restriction, uraemia-associated dysbiosis, and drugs [29,30,31]. Moreover, dietary restriction due to the high potassium content in most vitamin K-rich, green vegetables contributes to its deficiency [12]. Alongside dietary intake, vitamin K is recycled through the “vitamin K cycle”, which includes vitamin K epoxide reductase, DT-diaphorase and g-glutamylcarboxylase. Reduced recycling of vitamin K was found in rats with CKD, likely caused by the reduced activity of g-glutamyl-carboxylase, with a mechanism similar to the coumarins [32,33].

Patients affected by CKD are known to have increased risk of developing vascular calcification (VC) and bone fractures [34], which in turn contribute to the higher morbidity and mortality rate in CKD patients [34,35]. Several reports suggested that vitamin K2 may play a key role both in pathogenesis and prevention of those serious complications [36,37].

A cross-sectional observational study, the VIKI (Vitamin K Italian) study, evaluated the association between vitamin K reserves, vertebral fractures, and vascular calcifications, highlighting the prevalence of vitamin K deficiency in a setting of 387 hemodialysis patients. VK1 deficit resulted the strongest predictor of vertebral fractures (odds ratio [OR], 2.94; 95% confidence interval [CI], 1.38–6.26), while the deficiency of MK-4 was a predictor for aortic calcification (OR, 2.82; 95% CI, 1.14–7.01) [38].

Hemodialysis treatment, sevelamer (a phosphate binder) or vitamin K Antagonists (VKA) represent the major iatrogenic causes of vitamin K deficiency in patients with chronic renal failure [39]. CKD patients, including those on hemodialysis treatment, often use VKA drugs, especially for stroke prophylaxis in atrial fibrillation (AF). Warfarin, thus, can predispose to fragility fractures and vascular calcification through different mechanisms: directly, by inhibiting the carboxylation of osteocalcin (bone Gla protein or BGP) and other bone matrix proteins, and indirectly, because of the reduced dietary intake of foods rich in vitamin K in warfarin-users [40]. Currently, the non-vitamin K oral anticoagulants (NOACs) are being largely used to prevent stroke and cardioembolic complications in AF patients instead of vitamin K antagonists. However, their use in advanced CKD and end stage renal disease (ESRD) is to date contraindicated [41].

Indeed, Siontis et al., observed less bleeding with the standard dose (5 mg × 2/day) of apixaban than vitamin K inhibitor as well as a reduction in the risk of thromboembolism in a retrospective cohort study involving ESRD patients with AF [42].

Vitamin K2 reduced bioavailability due to phosphate binders (PBs) may be different according to the binder and to the type of menaquinone [39,43,44,45]. Neradova et al., verified the possible binding action of different PBs on vitamin K2 (MK-7) [44]. Calcium acetate/magnesium carbonate binds vitamin K2 regardless of the presence of phosphorus, lanthanum carbonate only in the absence of phosphorus, whereas sucroferric oxyhydroxide and sevelamer carbonate do not bind vitamin K2 in vitro [44]. Interestingly, a more recent investigation using a rat model by Neradova et al., showed that the combination of high vitamin K2 diet and PBs treatment significantly reduced VC, compared to MK7 or PBs treatment alone [40]. However, the use of sevelamer was significantly correlated with MK-4 deficiency, as well as warfarin administration [39]. The chemical reason has not yet been investigated, assuming that these are bonds mostly due to the form of the chelator [44]. On the other hand, a synergistic action of calcium mimetics and vitamin D analogues with vitamin K supplementation has been demonstrated more beneficial compared to the administration of each of these vitamins individually, with special reference to bone health [46]. Similar considerations can be applied to the complex setting of kidney transplantation (KT) patients [6]. In fact, impaired cardiovascular health related to vascular calcifications could be linked to a low vitamin K status also in KT recipients. An association between thoracic aorta calcification and shorter time on mycophenolate mofetil (MMF) treatment, an immunosuppressive agent, with current use of anti-vitamin-K has been previously suggested, confirming lower dp-ucMGP levels in KT patients receiving MMF therapy. This result is certainly also attributable to the improvement of the nutritional status and the greater contribution of the micronutrient [47].

### 4.1. Vitamin K: A Potential Role in the Development and Progression of CKD

Low peripheral vitamin K status has been previously associated with proteinuria and CKD stage [48]. The decarboxylated matrix protein Gla (dp-ucMGP) was used as an indirect marker for the determination of vitamin K concentrations on 3969 individuals with a mean age of 52.3 ± 11.6 years (48% male), enrolled in the “Prevention of Renal and Vascular end-stage Disease” [49,50]. The outcomes of this research were represented by the diagnosis of CKD (estimated Glomerular Filtration Rate (eGFR) <60 mL/min/1.73 m^2^) or the occurrence of microalbuminuria. During the 7.1 years of follow-up, 205 (5.4%) participants developed CKD and 303 (8.4%) developed microalbuminuria. For each doubling of plasma decarboxylated matrix protein Gla, the risk of onset of CKD and microalbuminuria was 1.85 [95% confidence interval (CI) 1.59–2.16, respectively; *p* < 0.001] and 1.19 (95% CI 1.07–1.32; *p* = 0.001), suggesting a possible prognostic value of dp-ucMGP in CKD, as it could imply a role for poor vitamin K status in the development of chronic renal failure [51]. In a recent analysis, it was already documented that both the deficiency of vitamin K and 25 OH-vitamin D, in almost equal measure, was associated with the progression of renal function decline and with the increased albumin/creatinine urinary excretion ratio [48]. Moreover, vitamins D and K have been suggested to cooperate in exerting favorable properties on bone protection, slowing VC progression, and in improving cardiovascular health [52].On the other hand, Kurnatowska et al., also highlighted a higher concentration of dp-ucMGP in patients with CKD, especially in stage V. The administration of vitamin K2 (90 mcg/day) resulted in a reduction in dp-ucMGP levels. Interestingly, plasma dp-ucMGP concentrations inversely correlated with eGFR and directly correlated with proteinuria and serum creatinine [53]. How vitamin K can be nephroprotective, also in reducing proteinuria, is still not fully understood and further evidences are needed.

### 4.2. CKD-MBD

Chronic kidney disease–mineral bone disorder (CKD-MBD) is a term used to identify the deterioration of bone quality and the consequent development of disorders in bone and mineral metabolism induced by impaired kidney function [54]. This inevitably places CKD patients, particularly those on hemodialysis, at higher risk of fracture, as compared to the general population [55].

Bone remodeling is a continuing dynamic process performed mainly by the two antagonistically acting cells, osteoblasts, which regulate the bone formation, and osteoclasts, responsible for the bone resorption process [56,57].

Several studies (both in vivo and in vitro) have proved that vitamin K is directly involved in bone metabolism. Some of these demonstrated that vitamin K2 inhibits bone resorption probably, in part due to the reduced production of bone resorbing substances including prostaglandin E2 and interleukin 6. It has also been shown that vitamin K is able to promote human osteoblast-induced bone mineralization in vitro, and to inhibit bone loss in steroid treated or ovariectomized rats [58]. Vitamin K2 is also a cofactor for some proteins involved in bone mineralization, namely osteocalcin (bone Gla protein or BGP) and matrix Gla protein (MGP) [59].

BGP is a small protein of 5.6 kDa, consisting of 49 amino acids, which is produced in bone by osteoblasts and minimally secreted into the circulation. As the matrix Gla protein, it is found in carboxylated and decarboxylated forms [60]; their serum levels are affected by age and hormonal status. Both forms increase with age, but after the menopause decarboxylated osteocalcin predominates [61]. It is mainly involved in the formation of hydroxyapatite and the consolidation of bone mass, but is shown to have various extraskeletal functions on glucose and energy metabolism, reproduction, and cognitive function [62]. In fact, it also stimulates the release of insulin by acting directly on the pancreas and indirectly inducing the secretion of glucagon-like peptide 1 (GLP-1) and adiponectin in the small intestine, taking part in glucose metabolism [63]. Interestingly, BGP has also been shown to stimulate angiogenesis and to upregulate nitric oxide (NO) signaling in endothelial cells, suggesting a protective role of this protein on reducing the risk of cardiovascular diseases [64,65].

MGP is a 10.6 kDa protein, consisting of 84 amino acids, insoluble in water. It is mainly synthesized by smooth muscle cells and chondrocytes and secreted into the extracellular matrix. It inhibits calcification and is only activated after the process of carboxylation and phosphorylation. Vitamin K, as a cofactor, facilitates its carboxylation at 5 glutamic acid residues at positions 2, 37, 41, 48, and 52 by γ-glutamyl carboxylase; in addition, 3 serine residues are phosphorylated at positions 3, 6, and 9 by casein kinase. The process of inhibiting vascular calcification would take place through the binding of calcium ions by carboxyl groups [66,67].

Gla-rich protein (GRP) has a molecular weight of 10.2 kDa and consists of 74 amino acids. Like other matrix proteins, GRP is vitamin K dependent and inhibits vascular calcifications, acting similarly to the matrix Gla protein, by binding and sequestrating calcium ions [68].

Growth Arrest Specific Protein 6 (GAS6) is a 75 kDa protein, activated in a vitamin K-dependent carboxylation process. GAS6 is mainly involved in the control of cell growth and proliferation and is secreted by osteoblasts to the bone matrix [69]. In detail, unlike the other VKDPs, GAS6 has been shown to increase osteoclast activity thus promoting bone resorption [70].

**Figure 1:**
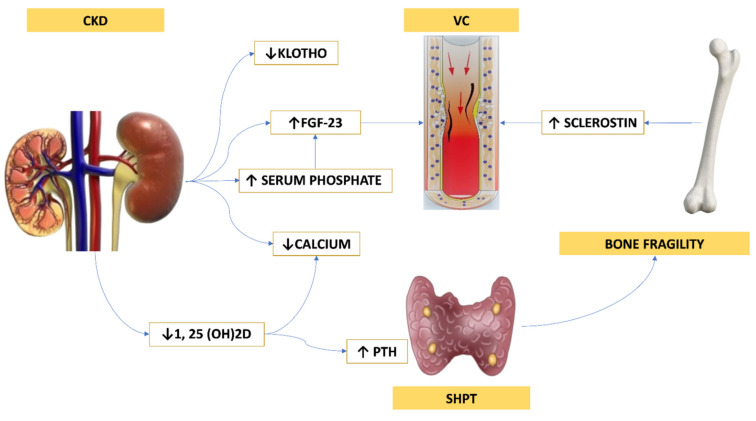
Complex interplay between kidney, parathyroid glands, bone and cardiovascular system in CKD-MBD pathogenesis. CKD is characterized by a secondary hyperparathyroidism: the progressive reduction of GFR leads to an increase of serum phosphate levels, a progressive hypocalcemia, and augmented FGF-23 production. Meanwhile, the reduced activity of the enzyme 1 alpha-hydroxylase induces a decrease of 1,25-dihydroxyvitamin D, further determining a PTH rising. High serum phosphate and FGF-23 levels also stimulate an increase of sclerostin production by osteocytes. Sclerostin and FGF-23 are involved in the progression of VC. Abbreviations: *CKD = Chronic Kidney Disease; FGF-23 = fibroblast growth factor 23; 1, 25 (OH)2D = 1,25-dihydroxy-vitamin D; PTH = parathyroid hormone; SHPT = Secondary hyperparathyroidism; VC = Vascular Calcification*.

In CKD, starting from stage IIIA, the impairment of bone tissue can present with high or low bone turnover, leading to a higher risk of fractures [71]. The different clinical pictures can be delineated in relation to parathyroid hormone (PTH) levels and bone turnover: hyperparathyroid osteopathy, or high turnover-osteopathy; this is characterized by secondary hyperparathyroidism, osteomalacia and osteoporosis, and adynamic bone disease (ABD), with the latter consisting of low PTH levels and decreased bone turnover, low bone volume but with normal mineralization, and markedly reduced cellularity with minimal or no fibrosis [72]. In addition to PTH, vitamin D, calcium and phosphorus, fibroblast growth factor-23 (FGF-23), sclerostin, and Klotho play a role in CKD-MBD (Figure 1) [71,73].

The increased bone sclerostin expression may also play a role in the improved FGF-23 expression, as it was proved to upregulate FGF-23 [74,75]. Furthermore, Bolignano et al., previously demonstrated that Cathepsin-K, a lysosomal cysteine protease secreted by activated osteoclasts and promoting bone and extracellular matrix remodeling, was associated with PTH levels, in a setting of 85 chronic hemodialysis patients, suggesting that this protein could represent a biomarker of CKD-MBD severity and PTH levels [76,77,78,79,80].

Vitamin K2 appears to be involved in this intriguing molecular interplay [24,81,82]. In research conducted on 210 women with osteoporosis, after six months treatment with vitamin K2, all indicators of metabolism and bone density had significantly increased, suggesting osteogenic activity. Others studies confirmed this evidence [83]. In addition, vitamin K2 counteracts osteoclastic activity. Rangel et al., demonstrated an increase in bone mass in ovariectomized mice supplemented with vitamin K [84]. On the same basis, 374 postmenopausal women with osteoporosis had more fractures with an impaired bone strength if they presented with vitamin K deficiency [85]. In a prospective work on 241 osteoporotic patients of both sexes, the administration of 45 mg/day of vitamin K2 resulted in a significant reduction in fractures [86]. An interesting question was also raised about a possible advantage from the vitamin K2-25OH vitamin D3 combination. Matsunaga et al., showed that the combined treatment seems more effective than single administrations in preventing bone loss on the femoral shaft and in the tibial metaphysis in ovariectomized rats [87]. Studies on the hemodialysis population are currently still few but all of the evidence suggest that vitamin K deficiency is an independent predictor of fracture risk [88,89,90].

### 4.3. Vitamin K and Hypertension in CKD

CKD patients with inadequate total vitamin K intake (both K1 and K2) had higher cardiovascular and all-cause mortality than those with adequate intake [91]. Vitamin K deficiency was acknowledged as independent predictor of cardiovascular disease (CVD) risk [92]. Moreover, Vitamin K2 deficiency or vitamin K functional inhibition by warfarin administration leads to calcium deposition in the arterial blood vessels [13]. Furthermore, vitamin K2 was shown to slightly increase HDL cholesterol and to decrease systemic inflammation [93,94]. Consistently, its supplementation could be supposed to slow vascular damage and prevent atherosclerosis, CVD and stroke [95,96,97,98]. Indeed, a connection between higher estimated menaquinone intake (above 21.6 μg/day) and decreased coronary heart disease-related mortality and aortic calcification was found, but there was no such correlation for phylloquinone [99,100,101]. In the PREVEND study, functional vitamin K deficiency was detected in 31% of the whole study population, and the incidence was much higher among the elderly and subjects with comorbidities, such as hypertension, type 2 diabetes, CKD, and cardiovascular disease [102]. Further results from ongoing interventional randomized clinical trials will better clarify if and what dosage vitamin K1 or K2 slows the progression of VC in CKD patients [103,104,105,106]. The international guidelines recommend the maintenance of arterial blood pressure values below 130/80 mmHg to reduce the cardio-renal risk in this type of patients [107]. Vitamin K2 seems to have a supporting role in the treatment of primary hypertension [108,109]. Liu Tian-Hao et al., also investigated the underlying mechanism by resorting to 16S rRNA sequencing, highlighting an influence of vitamin K2 on the complement system, calcium signals, and the renin-angiotensin-aldosterone system (RAAS) in an experimental model of salt-sensitive arterial hypertension [110]. The RAAS involvement in this model of salt-induced arterial hypertension was there confirmed and the administration of vitamin K2 was shown to exert an inhibitory effect on the RAAS-mediated pathways. In the same work, further analysis led to the identification of bacteria, including Dubosiella and Ileibacterium, favorably inducing RAAS modulation [111]. This finding also led to the assumption that probiotic compounds basing on these bacteria could be of help in improving metabolism and immunity also through the increased synthesis of vitamin K2 for the maintenance of endothelial functions [111,112].

To reinforce this thesis, Jensen et al., reported data on 79 hypertensive patients in Oakland showing how consumption of nattokinase-fermented soybean, rich in vitamin K2, is associated with beneficial changes in blood pressure (although diastolic blood pressure only achieved statistical significance, while for the systolic lowering a trend was suggested) [113]. Moreover, a case of hypotension has been reported after the administration of menaquinone [114]. Mansour et al., also showed how, implementing MK-7, mean arterial pressure (MAP) and peripheral diastolic blood pressure (DBP) decreasing [115]. However, blood pressure change on Vitamin K supplementation has been debated, and data are not consistent to date. In fact, observations did not confirm this blood pressure lowering effect [116,117,118,119,120,121]. Definitely, the pathophysiological link between vitamin K status and blood pressure control is not clearly established so far.

There are several hypotheses about phylloquinone (VK1). Phylloquinone, as well as vitamin K2, plays a key role in cardiovascular disease. Phylloquinone deficiency has been suggested as a risk factor for incident CVD in older treated hypertensives. Nevertheless, vitamin K1 intake is not seemingly associated with a reduced CVD risk. This could be due to the activities, mainly hepatic, of vitamin K1, whereas vitamin K2 exerts extrahepatic ones. However, vitamin K1 also seems to be involved in extrahepatic activities, and it was suggested to play a role in delaying the arterial stiffening when administered more than 2 mg/die, as well as in reducing vascular calcification [122,123,124], although Bellinge et al., failed to confirm an attenuation of arterial calcification activity through vitamin K1 administration [125]. Notwithstanding, vitamin K1 intake is nowadays out of interest due to its poor positive effects data. Furthermore, vitamin K2 effects appear to be stronger than vitamin K1, also due to its longer half-life and to higher concentrations in extrahepatic tissues of all K2 vitamers [28].

There is no evidence so far about the usefulness of vitamin K3 supplementation in CKD, arterial hypertension, and CVD. Furthermore, high-dose vitamin K3 has been questioned to be potentially toxic (e.g., liver damage, hemolytic anemia, etc.) [18], and its administration in humans is not worldwide recommended. However, vitamin K3 is easily and inexpensively produced, and it is very stable also because is not degraded by light, and low-dose could be used to treat vitamin K deficiency [18,126,127]. By the way, there are still not enough studies regarding its supplementation in these patients.

Hypertension is one of the first causes of AF [128,129]. In this cohort of patients, the risk of VC and CVD changes is also related to anticoagulant drug (VKAs vs. NOAC). Several clinical trials have reported that VKAs promote atherosclerotic calcification [97]. In this context, we must clearly look at the risk/benefit ratio.

These considerations are even more current in CKD patients. Indeed, in these people, there is an additional risk considering the coexistence of hypertension, CKD, and possible use of VKA (Table 1).

## 5. Discussion

**Figure 2:**
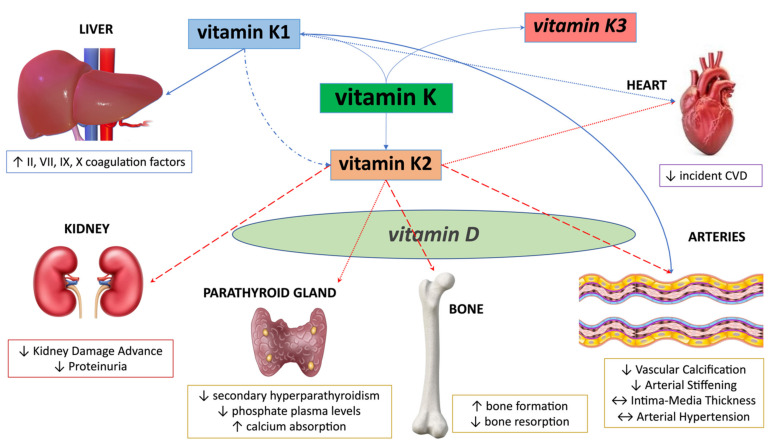
Vitamin K vitamers potential role on liver, kidney, parathyroid gland, bone, arteries, and heart. Potential synergism with vitamin D (on parathyroid, bone, and arteries) is also depicted.

In CKD patients, the parallel occurrence of impaired bone metabolism and CVD has been largely demonstrated; the latter are mostly promoted by VC, which in turn is determined by mineral dysregulation [130]. In detail, phosphate retention occurring in CKD concurs to the conversion of vascular smooth muscle cells to osteoblast-like cells producing bone matrix proteins which regulate arterial wall calcification [131]. Accumulating evidence suggest that this complex “calcification paradox” could be mediated also by vitamin K. Thus, the assessment of vitamin K concentration has been proven to be potentially of crucial importance in CKD patients, with an emerging role of this nutrient abs a marker for incident CVD, CKD development and progression, and consequent CKD-MBD. In this light, vitamin K supplementation should be recommended [52]. Since vitamin K2 has a longer half-life (days) than vitamin K1 (hours), it could be speculated that supplementation with vitamin K2, which is essential for extrahepatic VKDPs, could probably be cheaper [132]. Nevertheless, the use of vitamin K1 could be favorable because of its ability to transform into vitamin K2 but in doses 10 times higher than vitamin K2, so this field remains under debate [124]. Vitamin K toxicity has not been confirmed, and a tolerable upper limit for vitamin K has not been established so far [100], as already reported in the previous recommendations [133]. Although it has been questioned to be potentially toxic due to overcoagulation issues, very high dose intake has been suggested to be paradoxically associated to hypoprothrombinemia in rare human case reports [93,134]. Actually, a consensus about the daily dose needed to prevent the advance of VC or the incidence of fractures in CKD population has not been reached. Notwithstanding, 10 mg for vitamin K1 and 360 mcg/die until over 1080 mcg/die for MK-7 has been proposed as an adequate dosage [6]. So, vitamin K supplementation (especially menaquinone–vitamin K2) could have a protective role on both bone and cardiovascular health in patients with CKD. Indeed, a synergistic interplay has been suggested between vitamins D and K in exerting bone protection properties, as well as in slowing VC progression and in improving cardiovascular health [52]. However, no RCTs have been designed so far in order to explore the combined supplementation in CKD patients. Vitamin K vitamers potential role in affecting the liver, kidney, parathyroid gland, bone, arteries, and heart is depicted in Figure 2.

According to a Scientific Opinion provided by the European Food Safety Authority (EFSA), the vitamin K dietary reference values (DRVs) for the European population are estimated to be 1 µg/kg body weight per day of phylloquinone, which corresponds to an amount of 70 µg phylloquinone/day for adults, both women and men. Since data about menaquinones absorption, function and content in the body or organs are limited, EFSA released adequate intake recommendations for phylloquinone only [100]. This amount of phylloquinone could play a role in reduction of CVD progression, especially in Arterial Hypertension acting on arterial calcification activity and arterial stiffening [122,124,125]. There are still not studies about its role in CVD prevention. 

The National Institutes of Health (NIH) provides its recommendations for intake and administration of Vitamin K vitamers, through the Office of Dietary Supplements [135]. However, there is currently no definitive consensus on how to supplement vitamin K, whether with food or supplements. Few data about the bioavailability of different forms of vitamin K from food exist. The bacterial synthesis contributes in a small way to the production of menaquinones, but its exact support remains unclear [16]. Several multivitamins and/or multimineral supplements currently available contain vitamin K, alone or combined with other nutrients (calcium, magnesium, vitamin D), usually with a content of vitamin K less than 75% of the daily value [17,135]. Further randomized placebo-controlled trials using phylloquinone, menaquinones, or a combination of different vitamers are needed to confirm that maintaining a good vitamin K status could prevent fragility fractures and vascular calcifications in CKD.

## 6. Conclusions

This scoping review provides insights about the prevalence of functional vitamin K insufficiency and its clinical implications in CKD, particularly focusing on bone and CV health, as well as on arterial hypertension and the progression of kidney damage. According to our knowledge, the effect of vitamin K supplementation on arterial hypertension also in the hemodialysis population has never been extensively debated. Ongoing research suggests vitamin K as a new therapeutic approach, although the therapeutic dosage to obtain the benefits of supplementing this nutrient has not yet been firmly defined and further evidence is certainly needed. Hence, based on what emerged from our detailed review of the literature and the complex heterogeneity of the CKD population, a patient-centered strategy should be proposed.

## Figures and Tables

**Table 1:** Effects of supplementation of vitamin K and cardiovascular outcome.

Study	Sample Size and Type of Patients	Study Type	Vitamin K Assessment and/or Supplementation	Cardiovascular Outcome
Brandenburg et al. [123]	*n* = 72 patients with asymptomatic or mildly symptomatic AVC	12-month prospective, single-center, open-label, randomized interventional trial	VK1 2 mg/d *n* = 38 PL *n* = 34 for 12 months	Lower progression of AVC by 12% (*p* = 0.03) after VK1 vs. PL plasma dp-ucMGP by 45% (*p* < 0.001) in the VK1 group;
Geleijnse et al. [99]	*n* = 4807 Women and men aged ≥55 years without MI	prospective, population-based study (7–10 years)	diet rich in VK1 mean intake of VK1: <200 μg/d, 200–278 μg/d and >278 μg/d diet rich in VK2 mean intake of VK1: <21.6 μg/d, 21.6–32.7 μg/d and >32.7 μg/d	VK1—no association with incidents of CHD mortality, all-cause mortality and aortic calcification VK2—reduction of CHD mortality and inverse relation to all-cause mortality and severe aortic calcification
Braam et al. [96]	*n* = 181 Healthy postmenopausal Caucasians between 50 and 60 years of age (only female)	double-blind RCT	vitamin K_1_ (1 mg) + D_3_ (8 μg) supplementation	Distensibility (+8.8%, *p* < 0.05) Compliance (+8.6%, *p* < 0.05) Pulse pressure (−6.3%, *p* < 0.05) CCA elasticity (−13.2%, *p* < 0.01)
Shea et al. [36]	*n* = 489 hypertension patients under drug treatment	prospective longitudinal cohort study	K1 K2	Low k1 (<0.2 nmol/die) is risk factor for incident CVD in older men and women treated for hypertension but was not associated with CVD in those not treated for hypertension
Beulens et al. [126]	*n* = 564 postmenopausal women between 62 and 72 years of age (only female)	cross-sectional study	Dietary menaquinone intake (31.6 ± 12.3 mcg/d)	High dietary VK2 intake is associated with decreased coronary calcification
Knapen et al. [127]	*n* = 244 postmenopausal women (age 59.5 ± 3.3)	RCT	Menaquinone-7 supplementation (180 mcg/d)	Menaquinone-7 supplementation improved arterial stiffness in people with higher baseline stiffness index.
Vaccaro et al. [97]	*n* = 5296; age >50	cross-sectional study	Dietary phylloquinone intake (women, 90 mcg/d; men, and 120 mcg/d)	Inadequate dietary phylloquinone intake was a strong and significant predictor of higher arterial pulse pressure.

## Data Availability

Not applicable.
